# Multiple surveys employing a new sample‐processing protocol reveal the genetic diversity of placozoans in Japan

**DOI:** 10.1002/ece3.3861

**Published:** 2018-01-29

**Authors:** Hideyuki Miyazawa, Hiroaki Nakano

**Affiliations:** ^1^ Shimoda Marine Research Center University of Tsukuba Shimoda Shizuoka Japan

**Keywords:** distribution, genetic diversity, phylogeography, Placozoa, sampling method

## Abstract

Placozoans, flat free‐living marine invertebrates, possess an extremely simple bauplan lacking neurons and muscle cells and represent one of the earliest‐branching metazoan phyla. They are widely distributed from temperate to tropical oceans. Based on mitochondrial 16S rRNA sequences, 19 haplotypes forming seven distinct clades have been reported in placozoans to date. In Japan, placozoans have been found at nine locations, but 16S genotyping has been performed at only two of these locations. Here, we propose a new processing protocol, “ethanol‐treated substrate sampling,” for collecting placozoans from natural environments. We also report the collection of placozoans from three new locations, the islands of Shikine‐jima, Chichi‐jima, and Haha‐jima, and we present the distribution of the 16S haplotypes of placozoans in Japan. Multiple surveys conducted at multiple locations yielded five haplotypes that were not reported previously, revealing high genetic diversity in Japan, especially at Shimoda and Shikine‐jima Island. The observed geographic distribution patterns were different among haplotypes; some were widely distributed, while others were sampled only from a single location. However, samplings conducted on different dates at the same sites yielded different haplotypes, suggesting that placozoans of a given haplotype do not inhabit the same site constantly throughout the year. Continued sampling efforts conducted during all seasons at multiple locations worldwide and the development of molecular markers within the haplotypes are needed to reveal the geographic distribution pattern and dispersal history of placozoans in greater detail.

## 1 INTRODUCTION

**Figure 1:**
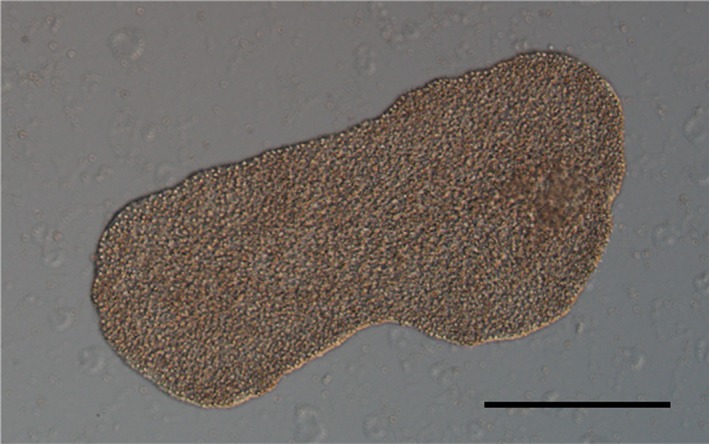
Photograph of Placozoa sp. H2, collected at Shimoda. Scale bar = 200 μm

Placozoans are free‐living marine invertebrates with small (<3 mm), asymmetric, disk‐like bodies composed of six cell types (Figure 1). Although no muscle or nerve cells have been identified in placozoans (Smith et al., 2014), their coordinated behavior during feeding suggests the existence of cell–cell communication (Smith, Pivovarova, & Reese, 2015). Asexual reproduction via binary fission and dispersive propagules has been observed in the laboratory (Thiemann & Ruthmann, 1991). Although embryogenesis after the 128‐cell stage has not been observed in placozoans (Eitel, Guidi, Hadrys, Balsamo, & Schierwater, 2011), the occurrence of sexual reproduction has been suggested based on the analysis of genetic recombination and the presence of sperm‐specific markers (Eitel et al., 2011; Signorovitch, Dellaporta, & Buss, 2005). Placozoa are one of the most basally branching lineages of metazoans, with recent phylogenomic studies suggesting that they are the sister group to a clade comprising Cnidaria and Bilateria (the phylogenetic position of Ctenophora remains controversial) (Borowiec, Lee, Chiu, & Plachetzki, 2015; Moroz et al., 2014; Pisani et al., 2015; Simion et al., 2017; Whelan, Kocot, & Halanych, 2015). Because of the lack of distinguishable morphological traits at the light microscopic level, *Trichoplax adhaerens* is still the only nominal species in the phylum Placozoa (Eitel, Osigus, DeSalle, & Schierwater, 2013). However, observations using electron microscopy revealed morphological differences among five groups cultured in the laboratory (Guidi, Eitel, Cesarini, Schierwater, & Balsamo, 2011), and differences in sensitivity to temperature and acidity within and between groups have also been recently reported (Schleicherová et al., 2016). Furthermore, molecular studies have elucidated considerable genetic diversity within the phylum (Pearse & Voigt, 2007; Signorovitch, Dellaporta, & Buss, 2006; Voigt et al., 2004). The genetic distances of mitochondrial 16S rRNA sequences between some placozoan groups have been reported to correspond to those between different families within Cnidaria and Porifera (Eitel & Schierwater, 2010; Voigt et al., 2004).

**Figure 2:**
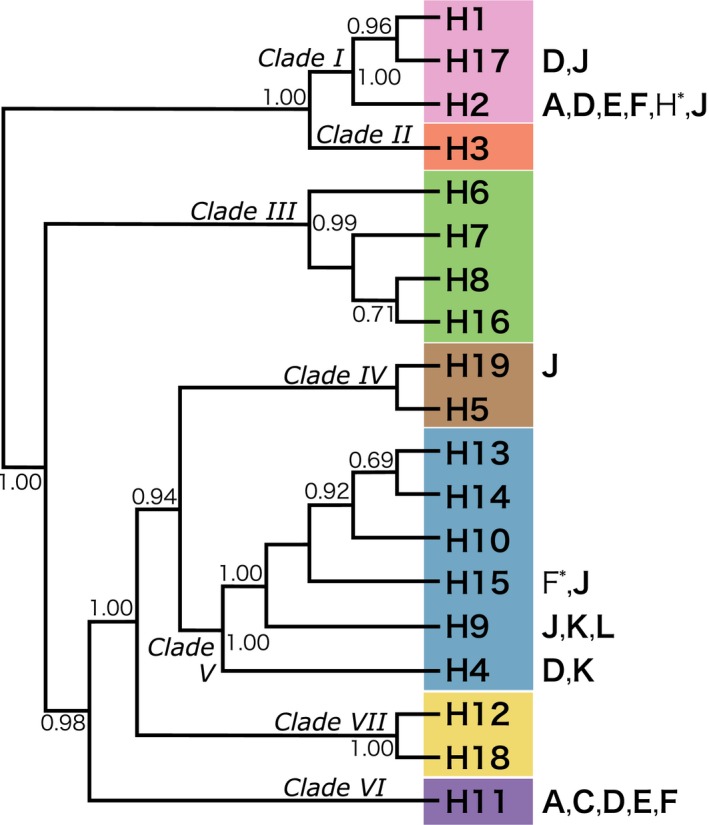
Cladogram of placozoan 16S haplotypes. Phylogenetic relationships, Bayesian posterior probabilities supporting nodes, clade names, and color codes are based on Eitel et al. (2013). For the haplotypes collected in Japan, the locations presented in Figure 3 are each shown on the right. Locations genotyped in previous studies: asterisks; those in this study: bold

Placozoans have been reported at 76 locations in temperate to tropical seas worldwide, and 19 haplotypes forming seven clades have been identified using 16S sequences thus far (see Figure 2; Eitel et al., 2013; Nakano, 2014). The number of existing haplotypes is estimated to be approximately 200 (Eitel & Schierwater, 2010; Eitel et al., 2013), and a habitat suitability model analysis suggested that placozoans are probably present in numerous nonsampled regions (Paknia & Schierwater, 2015). The current worldwide distribution map of placozoan 16S haplotypes is based on the genotyping results from 47 locations, but sampling has been performed only once at 38 of these locations (Eitel et al., 2013). Multiple surveys conducted in the Caribbean Sea from 2002 to 2004 and in Hong Kong from 2006 to 2007 have yielded multiple haplotypes from the same locations (Eitel & Schierwater, 2010; Signorovitch et al., 2006), leading the authors to believe that continued sampling during different seasons would also yield different haplotypes at a single location in temperate regions. In Japan, placozoans have been reported at nine locations in the three surrounding seas: the Sea of Japan, the Northern Pacific Ocean, and the East China Sea (Eitel & Schierwater, 2010; Nakano, 2014; Pearse, Uehara, & Miller, 1994; Pearse & Voigt, 2007; Sudzuki, 1977; Ueda, Koya, & Maruyama, 1999), and haplotypes collected at two locations have been reported: H15 for Shirahama, Wakayama (Miyazawa, Yoshida, Tsuneki, & Furuya, 2012) and H2 for Chatan, Okinawa (Eitel & Schierwater, 2010).

Placozoans have been sampled from shallow (<20 m depth) seawater environments using two distinct methods (Maruyama, 2004): (1) In slide sampling, glass or plastic slides are placed in natural seawater and are generally retrieved after more than 10 days. Placozoans on the slides are collected under a stereomicroscope. (2) In substrate sampling, natural substrate materials such as stones and molluscan shells are collected together with ambient seawater inside a container. There are several protocols for subsequent processing of the substrates after collection. One method for processing these substrates, a process we refer to as “passive substrate sampling,” is to place the substrates and seawater into a tank and collect the animals from the walls of the tank (Miyazawa et al., 2012). Slides may also be placed in the tank, and placozoans on the slides can then be collected under a stereomicroscope (Miyazawa et al., 2012). Another method is what we refer to as “agitation substrate sampling,” in which the container with the substrate and seawater is vigorously shaken for several seconds to detach placozoans from substrates. The seawater is then decanted into Petri dishes, and placozoans are collected from the Petri dishes under a stereomicroscope. Slide sampling requires special equipment to prevent the submerged glass slides from being carried away or broken by the current and requires a minimum of approximately 10 days to collect the animals. Passive substrate sampling generally requires a few days, as it takes time for the animals to migrate from the substrates to the walls or slides. Agitation substrate sampling can yield placozoans at multiple locations in a single day, but the collected placozoans are frequently damaged as a result of the shaking process (Maruyama, 2004).

In a previous study, we identified placozoans at all six surveyed locations in Japan, suggesting a wide distribution of placozoans around Japan and in the Northern Pacific Ocean (Nakano, 2014). Here, we introduce a new processing protocol for substrate sampling referred to as “ethanol‐treated substrate sampling,” which can be performed at multiple locations in a single day and does not appear to damage placozoans. We succeeded in collecting placozoans using this protocol in three new sampling locations: Shikine‐jima Island, Chichi‐jima Island, and Haha‐jima Island. We further determined the 16S rRNA sequences of the placozoans from eight locations within Japan, and we report five haplotypes that have not been previously described in the country. On Shikine‐jima Island, we performed slide and ethanol‐treated substrate sampling from 2015 to 2016, yielding multiple haplotypes, including haplotypes that were not found in other areas of Japan. Our results show that the haplotype distribution at a site varies with the seasons, and we propose that sampling efforts conducted during different seasons at multiple locations worldwide, together with analyses of molecular markers to reveal the genetic structure within haplotypes, are essential for revealing the distribution and dispersal history of placozoans.

## 2 MATERIALS AND METHODS

### 2.1 Sampling methods

In preliminary experiments, when an approximately 5% volume of 99.5% ethanol (Wako, Osaka, Japan) was added to seawater in glass dishes containing placozoans, the bodies of the animals shrunk and detached from the bottom after slight shaking of the dish. The animals drifted without any movement and showed no reaction when disturbed by the water current generated using a micropipette. Three minutes later, all of the animals were transferred to new dishes containing seawater without ethanol. Thereafter, the placozoans attached to the bottom and began to move around, with their survival being confirmed after 24 hr. The experiment was repeated three times, with all animals in all experiments surviving after 24 hr. This finding suggests that exposure to seawater containing approximately 5% ethanol for <3 min causes little damage to placozoans. Based on these preliminary experiments, a new processing protocol for substrate sampling that causes no apparent harm to the animals was developed. The new processing protocol is as follows: (1) Stones, shells, and ambient seawater are collected in containers; (2) 99.5% ethanol is added to each container to achieve an ethanol concentration of approximately 5%; (3) the tubes are shaken gently for approximately 5s; (4) the suspended seawater is decanted into glass dishes; (5) placozoans are collected from the dishes and transferred to different new containing seawater without ethanol. This new processing protocol, referred to hereafter as “ethanol‐treated substrate sampling,” was successfully tested along a rocky shore in Shimoda (Table 1).

**Table 1:** Locations where placozoans have been collected in Japan

Location	Locations in Figure 3	Sites	Habitat type	Sampling and processing method	Date collected (year/month)	Citation of collection	Haplotype and collected specimens		Accession number	Citation of genotyping
Noto, Ishikawa	A		Rocky shore	Agitation	2012/10	Nakano (2014)	H2	: 1	LC306931	This study
			Rocky shore	Agitation	2012/10	Nakano (2014)	H11	: 2	LC306930	This study
Oki, Shimane	B		–	–	–	Pearse and Voigt (2007)	–		–	
Tateyama, Chiba	C		Outdoor tank	Slide	2012/11	Nakano (2014)	H11	: 2	LC306945	This study
Shimoda, Shizuoka	D	Figure 3 Db	–	Slide	1977/2, 1977/6	Sudzuki (1977)	–		–	
		Figure 3 Da	Outdoor tank	Slide	2010/10	Nakano (2014)	H4	: 1	LC306940	This study
		Figure 3 Da	Outdoor tank	Slide	2012/1	Nakano (2014)	H2	: 1	LC306939	This study
		Figure 3 Da	Outdoor tank	Slide	2012/1	Nakano (2014)	H2	: 5	LC322278	This study
		Figure 3 Da	Outdoor tank	Slide	2012/4	Nakano (2014)	H2	: 1	LC322279	This study
		Figure 3 Da	Outdoor tank	Slide	2012/5	Nakano (2014)	H11	: 1	LC306937	This study
		Figure 3 Da	Outdoor tank	Slide	2012/7	Nakano (2014)	H2	: 8	LC322280	This study
		Figure 3 Da	Rocky shore	Ethanol	2014/9	This study	–		–	
		Figure 3 Da	Outdoor tank	Slide	2014/9	This study	H2	: 2	LC336424	This study
		Figure 3 Da	Outdoor tank	Slide	2014/12	This study	H11	: 2	LC336422	This study
		Figure 3 Da	Outdoor tank	Slide	2015/2	This study	H2	: 1	LC336425	This study
		Figure 3 Da	Outdoor tank	Slide	2015/5	This study	H2	: 2	LC336426	This study
		Figure 3 Da	Outdoor tank	Slide	2015/6	This study	H2	: 2	LC336427	This study
		Figure 3 Da	Outdoor tank	Slide	2015/6	This study	H11	: 1	LC336423	This study
		Figure 3 Da	Outdoor tank	Slide	2015/6	This study	H17	: 1	LC306938	This study
Sugashima, Mie	E		Outdoor tank	Slide	2012/11	Nakano (2014)	H2	: 2	LC306944	This study
			Outdoor tank	Slide	2012/11	Nakano (2014)	H11	: 5	LC306943	This study
			Rocky shore	Passive	2016/1	This study	H11	: 4	LC322281	This study
Shirahama, Wakayama	F		–	–	–	Ueda et al. (1999)	–		–	
		Figure 3 Fa	–	Slide	1989/11–1992/12	Maruyama (2004)	–		–	
		Figure 3 Fa	–	Agitation	1990/7–1992/12	Maruyama (2004)	–		–	
		Figure 3 Fb	Rocky shore	Passive	2007/9	Miyazawa et al. (2012)	H15	: 1	NC_015309	Miyazawa et al. (2012)
		Figure 3 Fc	Boat dock	Slide	2012/12	Nakano (2014)	H2	: 7	LC306942	This study
		Figure 3 Fc	Boat dock	Slide	2012/12	Nakano (2014)	H11	: 1	LC306941	This study
Sesoko, Okinawa	G		–	Slide	1994	Pearse et al. (1994)	–		–	
			Rocky shore	Agitation	2012/6	Nakano (2014)	–		–	
Chatan, Okinawa	H		Boat dock	Slide	2007/3	Eitel and Schierwater (2010)	H2	: 2	GQ901119, GQ901120	Eitel and Schierwater (2010)
Iriomote, Okinawa	I		–	–	1989	Pearse and Voigt (2007)	–		–	
Shikine‐jima Island, Tokyo	J	Figure 4c	Rocky shore	Ethanol	2015/5	This study	H17	: 29	LC306933	This study
		Figure 4a	Rocky shore	Ethanol	2015/8	This study	H2	: 4	LC306935	This study
		Figure 4a	Rocky shore	Ethanol	2015/8	This study	H19	: 2	LC306934	This study
		Figure 4a	Rocky shore	Ethanol	2015/9	This study	H2	: 9	LC322276	This study
		Figure 4a	Rocky shore	Ethanol	2015/9	This study	H9	: 10	LC306936	This study
		Figure 4a	Rocky shore	Ethanol	2015/9	This study	H15	: 1	LC306932	This study
		Figure 4d	Stony beach	Ethanol	2015/9	This study	H9	: 2	LC322277	This study
		Figure 4d	Stony beach	Ethanol	2015/9	This study	H15	: 1	LC322274	This study
		Figure 4e	Rocky shore	Slide	2016/2	This study	H17	: 10	LC322275	This study
Chichi‐jima Island, Tokyo	K	Figure 5b	Boat dock	Ethanol	2016/6	This study	H4	: 3	LC306927	This study
		Figure 5a	Rocky shore	Ethanol	2016/6	This study	H9	: 1	LC306928	This study
Haha‐jima Island, Tokyo	L	Figure 6a	Rocky shore	Ethanol	2016/6	This study	H9	: 3	LC306929	This study
		Figure 6b	Rocky shore	Ethanol	2016/6	This study	H9	: 4	LC322273	This study

Agitation, agitation substrate sampling; passive, passive substrate sampling; ethanol, ethanol‐treated substrate sampling; slide, slide sampling.

**Figure 3:**
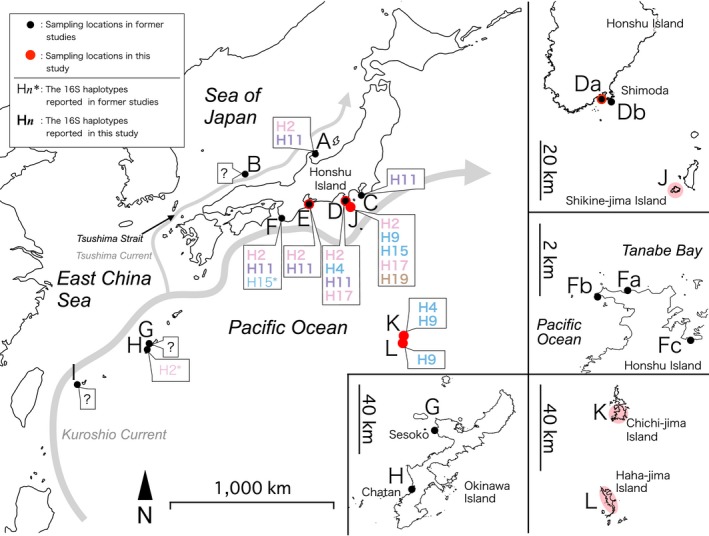
Distribution of 16S haplotypes in Japan. Locations where placozoans were collected in previous studies: black circles; collection locations of this study: red circles. 16S haplotypes of placozoans genotyped in previous studies: asterisks; those from this study: bold. The colors of the haplotypes are based on the color codes presented in Figure 2. Map source: Natural Earth (http://www.naturalearthdata.com/) and Geospatial Information Authority of Japan (http://www.mlit.go.jp/kokudoseisaku/kokudojoho.html)

**Figure 4:**
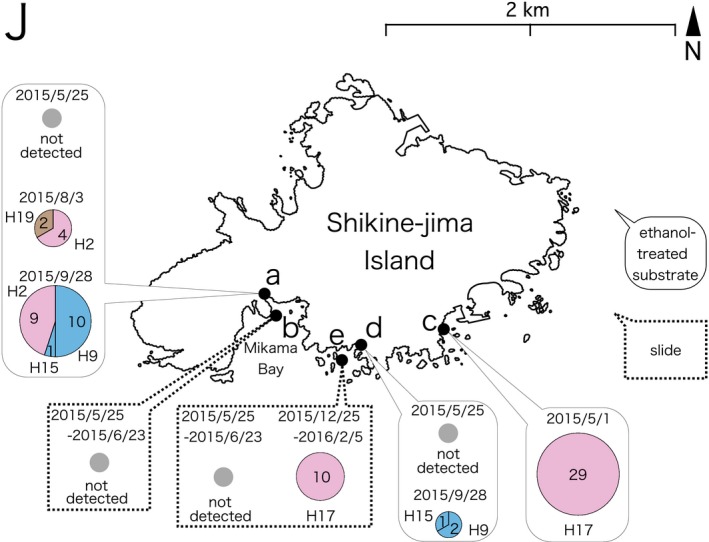
16S haplotype distribution and sampling dates (year/month/day) on Shikine‐jima Island. The results of ethanol‐treated substrate sampling and slide sampling are presented in rounded rectangles and dotted‐line rectangles, respectively. The radii of the circle graphs in the results are proportional to the square root of the sample size. The colors of haplotypes are based on the color codes presented in Figure 2. Map source: Geospatial Information Authority of Japan (http://www.mlit.go.jp/kokudoseisaku/kokudojoho.html)

Ethanol‐treated substrate sampling was performed on Shikine‐jima Island and the Bonin Islands (Ogasawara Islands), where placozoans have not been reported previously. Stones, shells, and ambient seawater were collected by hand from different depths of down to approximately 50 cm and placed into 50‐ml centrifuge tubes (27 mm inside diameter; Ina‐Optika, Osaka, Japan) along the rocky shore during low tide. Five to 10 centrifuge tubes were used at each location. Slide sampling was also conducted on Shikine‐jima Island, where slide glasses (S9213, 76 mm × 52 mm; Matsunami, Osaka, Japan) in staining dishes (Microscope Slide Staining Dish; Kartell, Melbourne, Australia) were placed on concrete blocks fixed to the sea bottom at two sites: J‐b (10 m depth) and J‐e (5 m depth) (Figures 3 and 4). The observation and collection of placozoans were carried out using a light stereomicroscope (SZX7; Olympus, Tokyo, Japan) and a micropipette (P200; Gilson, Villiers, France).

Permission to collect the substrates and seawater in the Ogasawara National Park was obtained from the Ogasawara Ranger Office for Nature Conservation, Ministry of the Environment, Japan.

See Nakano (2014) for details of the sampling conducted at Noto, Tateyama, Shimoda, Sugashima, and Shirahama.

### 2.2 DNA extraction and PCR conditions

Fixation and DNA extraction were performed for all placozoans collected from 2010 to 2012 at Noto, Tateyama, Shimoda, Sugashima, and Shirahama using FTA Elute Micro Cards (GE Healthcare, Milwaukee, WI, USA) according to Eitel and Schierwater (2010).

The animals collected from 2014 to 2016 from Shimoda, Sugashima, Shikine‐jima Island, Chichi‐jima Island, and Haha‐jima Island were individually transferred to 1.5‐ml centrifuge tubes using a micropipette under a microscope. For the samples from Shikine‐jima Island, the genomic DNA of each collected placozoan was extracted on the day of collection. Placozoans collected on the Bonin Islands were stored in 170 μl of 99.5% ethanol with 30 μl of seawater at room temperature for up to 11 days before DNA extraction. Genomic DNA was extracted using the Wizard Genomic DNA Purification Kit (Promega, Madison, WI, USA) following the manufacture's protocol (Isolation of Genomic DNA from Animal Tissue and Tissue Culture Cells).

The mitochondrial 16S rRNA of individuals was amplified via PCR using the forward primer 5′–GTTAATTGCTGGCCTGTATG–3′ (Voigt et al., 2004) or 5′ ‐CGAGAAGACCCCATTGAGCTTTACTA‐3′ (Signorovitch et al., 2006) and the reverse primer 5′–TACGCTGTTATCCCCATGGTAACTTT–3′ (Signorovitch et al., 2006). PCR was performed using ExTaq, SapphireAmp Fast PCR Master Mix, or EmeraldAmp PCR Master Mix (all Takara Bio, Otsu, Japan) with a LifeECO Thermal Cycler (Bioer Technology, Hangzhou, China), a T‐100 Thermal Cycler, or an MJ Mini Thermal Cycler (both Bio‐Rad, Laboratories, Hercules, CA, USA). The PCR conditions were generally as follows: 94°C denaturation for 1 min; 35 cycles of 98°C for 10 s, 55°C for 30 s, and 72°C for 1 min; and a final extension at 72°C for 1 min. The PCR products were purified using exonuclease I and alkaline phosphatase (Calf intestine) (both Takara Bio, Otsu, Japan) or the QIAquick PCR purification kit (Qiagen, Hilden, Germany). The DNA sequencing of the purified PCR products was outsourced to FASMAC (Atsugi, Japan). 16S rRNA sequences were deposited in GenBank, with accession numbers LC306927–LC306945, LC322273–LC322281, and LC336422–LC336427 (Table 1).

## 3 RESULTS

**Figure 5:**
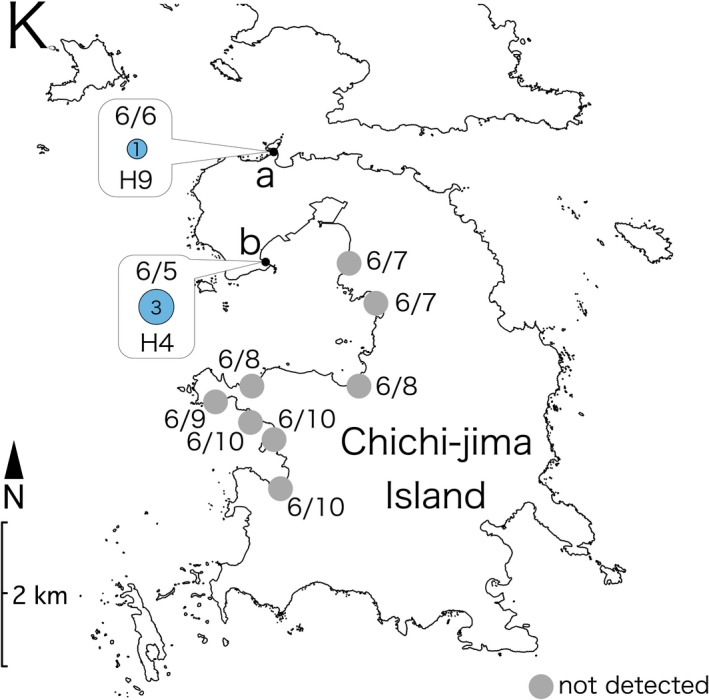
16S haplotype distribution and sampling dates (month/day) on Chichi‐jima Island. The radii of the circle graphs in the results are proportional to the square root of the sample size. Sites where no placozoans were detected are indicated with gray‐filled circles. All dates are in 2016. The colors of the haplotypes are based on the color codes presented in Figure 2. Map source: Geospatial Information Authority of Japan (http://www.mlit.go.jp/kokudoseisaku/kokudojoho.html)

**Figure 6:**
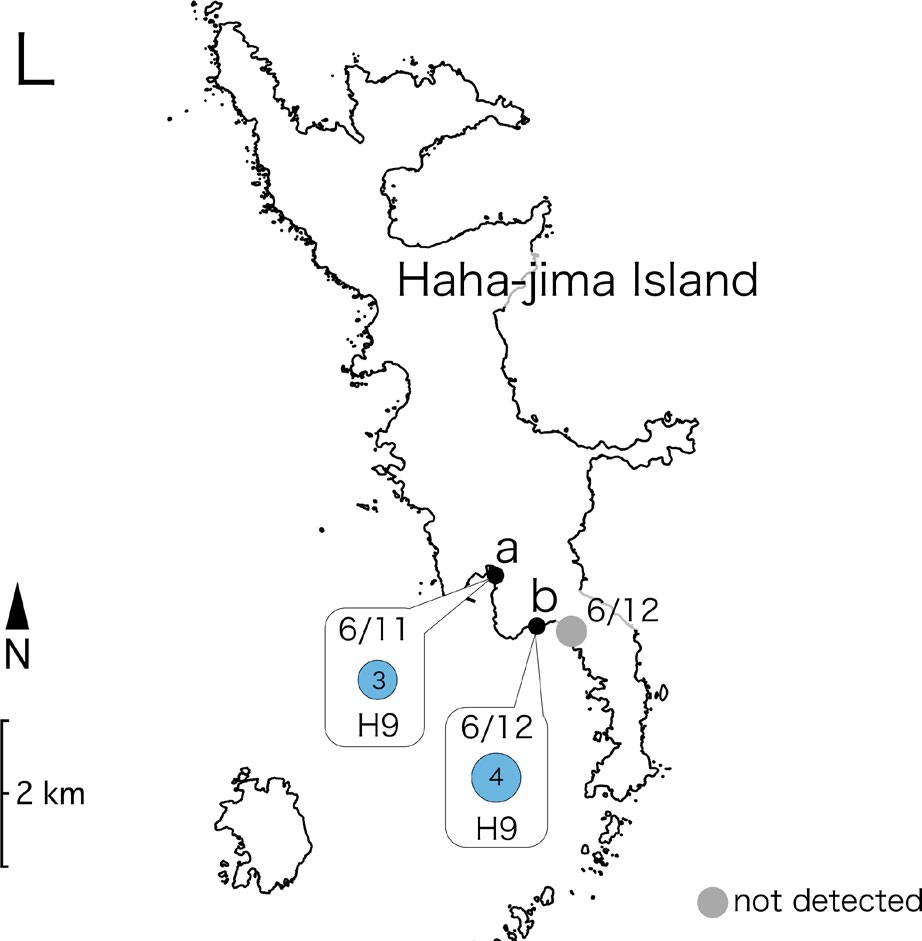
16S haplotype distribution and sampling dates (month/day) on Haha‐jima Island. The radii of the circle graphs in the results are proportional to the square root of the sample size. The sites where no placozoans were detected are indicated with gray‐filled circles. All dates are in 2016. The colors of the haplotypes are based on the color codes presented in Figure 2. Map source: Geospatial Information Authority of Japan (http://www.mlit.go.jp/kokudoseisaku/kokudojoho.html)

Ethanol‐treated substrate sampling performed at three sites on Shikine‐jima Island (Figure 3J) yielded 58 placozoans, and slide sampling at two sites resulted in 10 collected specimens (Figure 4). On Chichi‐jima Island (Figure 3K) and Haha‐jima Island (Figure 3L), four and seven specimens were isolated by ethanol‐treated substrate sampling, respectively (Figures 5 and 6).

The mitochondrial 16S genotyping of placozoans collected in Japan is summarized in Figures 2 and 3 and Table 1. H2 and H11 placozoans showed a wide distribution around Japan, with both haplotypes being found along the coasts of the Pacific Ocean and the Sea of Japan (Figure 3). H11 placozoans have previously only been collected in Monterey Bay (California, USA) (Pearse & Voigt, 2007, 2010); nevertheless, placozoans of this haplotype were found at five locations around Japan.

The haplotypes of the placozoans collected on Shikine‐jima Island were H2, H9, H15, H17, and H19 (Figure 4). As H17 and H19 placozoans have only been reported in Monterey Bay (California, USA) and Adelaide (Australia), respectively, the specimens collected on Shikine‐jima Island represent the second report of these haplotypes worldwide. On the Bonin Islands (Chichi‐jima and Haha‐jima Islands; Figure 3K,L), H4 and H9 placozoans were isolated (Figures 5 and 6). As H9 placozoans have previously been found only in Turkey (Eitel & Schierwater, 2010), Australia (Pearse & Voigt, 2007), and Bermuda (Signorovitch et al., 2006), this work constitutes the first report of H9 placozoans from the Northern Pacific Ocean.

Our sampling efforts resulted in the collection of 79 placozoans from three previously unreported locations (Shikine‐jima, Chichi‐jima, and Haha‐jima Islands), increasing the number of locations from which these animals have been collected to 12 in Japan and 79 worldwide. In addition to the two haplotypes previously reported from Japan (H2 and H15) (Eitel & Schierwater, 2010; Miyazawa et al., 2012), five haplotypes (H4, H9, H11, H17, and H19) were newly collected in Japan in the present study. No new haplotypes that did not belong to the 19 known haplotypes were detected in our surveys.

Concerning habitat type, sampling at boat docks yielded three haplotypes (H2, H4, and H11); sampling at outdoor tanks yielded four (H2, H4, H11, and H17); sampling along rocky shores yielded six (H2, H9, H11, H15, H17, and H19); and sampling on stony beaches yielded two (H9 and H15) (Table 1).

## 4 DISCUSSION

Six sampling efforts conducted using ethanol‐treated substrate sampling on Shikine‐jima Island yielded a total of 58 placozoans (Figure 4), showing that ethanol‐treated substrate sampling is an efficient protocol for collecting placozoans from natural environments. However, 13 sampling efforts performed on the Bonin Islands yielded only 11 placozoans (Figures 5 and 6). We are of the opinion that this disparity resulted not from the sampling method but from the biological nature of placozoans. The success of placozoan collection has been reported to depend largely on the weather and the microenvironment at collection sites (Nakano, 2014; Paknia & Schierwater, 2015; Pearse & Voigt, 2007). In the present study, the number of collected placozoans from site J‐a on Shikine‐jima Island fluctuated with the sampling date (Figure 4). Therefore, we consider ethanol‐treated substrate sampling to be an efficient collection protocol, and we expect that performing ethanol‐treated substrate sampling on different dates and at different sites on the Bonin Islands will yield more placozoans.

As more placozoans are collected from slides suspended above the sea bottom than those placed on the sea bottom, it has been suggested that a pelagic phase of placozoans exists (Pearse & Voigt, 2007). For example, swarmers, which bud off from the dorsal surface of individuals, have been reported to float in seawater (Thiemann & Ruthmann, 1991). Embryos released by adults have also been suggested to be planktonic (Eitel et al., 2011). Additionally, it is likely that adults that detach from the substrate are easily carried away by ocean currents. In Japan, the warm Kuroshio Current originates from east of the Philippines and reaches the Pacific coast of the country via Taiwan (Figure 3). In the Sea of Japan, the Tsushima Current, a branch of the Kuroshio Current, flows along the Japanese coast from the south through the Tsushima Strait (Figure 3). It has been reported that the Kuroshio and the Tsushima Currents impact the distribution of various animals, including sponges, clams, mantis shrimp, sunfish, and halfbeaks (Cheng & Sha, 2017; Hoshino, Saito, & Fujita, 2008; Yamada, Ishibashi, Toyoda, Kawamura, & Komaru, 2014; Yoshita et al., 2009; Yu, Kai, & Kim, 2016). H2 placozoans have been reported from Okinawa and Hong Kong (Eitel & Schierwater, 2010; Pearse & Voigt, 2007) and were collected on the coasts of both the Pacific and the Sea of Japan during this study (Figure 3). Therefore, the Kuroshio and Tsushima Currents may account for the wide distribution of H2 placozoans in Japan. The isolation of H11 placozoans from the coasts of both the Sea of Japan and the Pacific also suggests the effects of the two currents on their distribution, and conducting further collections at Okinawa, Taiwan, Hong Kong, or the Philippines may reveal H11 placozoans at those locations where the Kuroshio Current flows. H15 placozoans have been found in Boracay (Philippines) (Eitel & Schierwater, 2010), in Shirahama (Honshu Island) (Miyazawa et al., 2012, and this study) and in Shikine‐jima Island (this study), suggesting that the Kuroshio Current has an effect on the dispersal of this haplotype. Performing further collections on the coast of the Sea of Japan could reveal the effects of the Tsushima Current on its distribution.

H4 placozoans were shared between Shimoda and Chichi‐jima Island, but only one H4 placozoan was collected during our continuous survey conducted in Shimoda from 2010 to 2015 (Table 1). Furthermore, despite the wide distribution of H2, H11, and H15 placozoans along the Pacific coast of Japan, no H2, H11, or H15 placozoans were found on the Bonin Islands. Ethanol‐treated substrate sampling was performed in a similar environment (shallow water along a rocky shore) on both the Shikine‐jima and Bonin Islands, but haplotypes collected at Shikine‐jima, such as H2, H15, H17, and H19, were not found on the Bonin Islands. These results suggest that successful dispersal of placozoans between the Honshu/Shikine‐jima Islands and the Bonin Islands may be rare or absent. The Izu Islands stretch between Honshu Island and the Bonin Islands, and carrying out collections on these islands will illustrate the extent of both northward and southward dispersal and may clarify the reason for the lack of dispersal between Honshu Island and the Bonin Islands.

**Figure 7:**
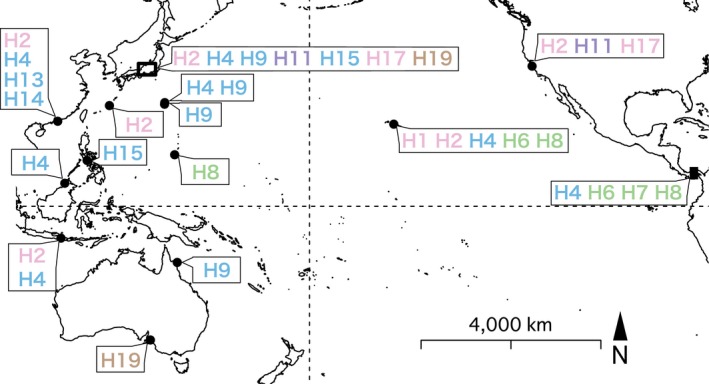
16S haplotype distribution in the Pacific Ocean. Multiple sampling locations on the Honshu/Shikine‐jima Islands and in Panama are shown with rectangles. The colors of the haplotypes are based on the color codes presented in Figure 2. Map source: Natural Earth (http://www.naturalearthdata.com/)

The 16S haplotypes of placozoans reported from the Northern Pacific Ocean, including Japan, are diverse (Figure 7), and it is difficult to estimate the dispersal routes of each haplotype based on its known distribution. For example, H9 and H19 placozoans are present in Japan and Australia but have not been found at intermediate locations where placozoans have been genotyped, such as Guam (USA), Boracay (Philippines), Sabah (Malaysia), and Bali (Indonesia) (Eitel et al., 2013). As different haplotypes were found in the samplings performed in different months on Shimoda and Shikine‐jima Island (Figure 4; Table 1), the haplotypes at a given site may not be constant throughout the year. Continued sampling will be necessary to elucidate the genetic diversity of placozoans at each site, and new samplings at previously 16S‐genotyped sites during different seasons may yield different haplotypes.

There were some differences between the habitat types where the specimens of certain haplotypes were collected in this study and in previous studies. For example, H4 and H9 were sampled from an outdoor tank and a rocky shore, respectively, in this study and have not been previously reported at such sites (Table 1). H19, which was previously collected once on a stony beach (Eitel et al., 2013), was collected on a rocky shore in this study (Table 1). H11 and H17 have been reported only from tanks at the Monterey Bay Aquarium (California, USA) (Pearse & Voigt, 2007, 2010). In this study, H11 was collected on a boat dock, from an outdoor tank and on a rocky shore, and H17 was also collected from an outdoor tank and on a rocky shore (Table 1), suggesting that H11 and H17 are present in various environments. These results suggest that haplotypes that have previously only been reported in certain environments may be found in other environments with further sampling.

An effect of weather on the number of collected placozoan individuals has been previously suggested (Nakano, 2014; Pearse & Voigt, 2007, 2010). In the present study, multiple samplings during different seasons resulted not only in a change in the number of collected specimens, but a change in the collected 16S haplotypes, especially for those from Shimoda and Shikine‐jima Island (Figure 4; Table 1). This observation suggests that the 16S haplotypes found at a certain site might also easily change, depending on the weather and other environmental conditions.

In previous studies, increasing the number of sampling locations has resulted in the discovery of new haplotypes, and it has been estimated that more than 200 haplotypes exist worldwide (Eitel & Schierwater, 2010; Eitel et al., 2013; Pearse & Voigt, 2007, 2010). However, we failed to collect new haplotypes among our samples. Our sampling was mostly performed in outdoor tanks and along rocky shores (Table 1), all at depths shallower than 50 cm. These conditions are similar to those of previous studies sampling placozoans in Japan. Further sampling efforts in other environments where placozoans have not been previously reported in Japan, such as open ponds, mangroves, or algae beds (Eitel et al., 2013), or at a greater depth, may be needed to obtain new haplotypes from Japan.

A recent study revealed that population growth rates of placozoans are negatively affected by acidity stress (Schleicherová et al., 2016). In Mikama Bay, on Shikine‐jima Island, where sites J‐a and J‐b are located (Figure 4), shallow CO_2_ seeps result in reduced pH zones (Agostini et al., 2015). No placozoans were collected from J‐b, whereas 26 were collected from J‐a. Collection at J‐a was performed in tide pools separated from the open sea at low tide, and the shallow CO_2_ seeps are expected to have little impact on the pH of the tide pools; thus, it is assumed that the placozoans that entered the tide pool were able to proliferate. Moreover, slides placed at J‐b were constantly exposed to a reduced pH, probably resulting in the failure of collection. The effect of low pH has been reported only in laboratory‐cultured placozoans (Schleicherová et al., 2016). Further studies at multiple sites exhibiting various pH levels within Mikama Bay would illustrate the effects of long‐term exposure to a reduced pH on wild placozoans and the ecological differences among the 16S haplotypes.

Our results revealed high genetic diversity of placozoans around Japan, especially in Shimoda and on Shikine‐jima Island, and showed that the distribution of placozoan 16S haplotypes varies according to the season. The Kuroshio and Tsushima Currents may have an effect on the dispersal of placozoans around Japan, but the dispersal history of placozoans in the Northern Pacific Ocean is far from certain. Because it is impossible to elucidate the dispersal routes of widely distributed 16S haplotypes based solely on 16S sequences, the development of reliable molecular markers within the haplotypes with a population‐level resolution is essential. Moreover, to reconstruct the phylogeographic patterns of Placozoa, further sampling efforts conducted not only at many locations but also in various environments and during different seasons are needed.

## DATA ACCESSIBILITY

DNA sequences: GenBank accessions LC306927–LC306945, LC322273–LC322281, and LC336422–LC336427.

## CONFLICT OF INTEREST

None declared.

## AUTHOR CONTRIBUTIONS

H.M. and H.N. designed the project, performed the research, analyzed the data, and wrote the manuscript.
